# Internal Motion Estimation by Internal-external Motion Modeling for Lung Cancer Radiotherapy

**DOI:** 10.1038/s41598-018-22023-3

**Published:** 2018-02-27

**Authors:** Haibin Chen, Zichun Zhong, Yiwei Yang, Jiawei Chen, Linghong Zhou, Xin Zhen, Xuejun Gu

**Affiliations:** 1 School of Biomedical Engineering, Southern Medical University, Guangzhou, 510515 China; 2 Department of Computer Science, Wayne State University, Detroit, MI 48202 USA; 3 Department of Physics, Zhejiang Cancer Hospital, Zhejiang Key Laboratory of Radiation Oncology, Hangzhou, 310022 China; 4 Department of Radiation Oncology, The University of Texas, Southwestern Medical Center, Dallas, Texas 75390 USA

## Abstract

The aim of this study is to develop an internal-external correlation model for internal motion estimation for lung cancer radiotherapy. Deformation vector fields that characterize the internal-external motion are obtained by respectively registering the internal organ meshes and external surface meshes from the 4DCT images via a recently developed local topology preserved non-rigid point matching algorithm. A composite matrix is constructed by combing the estimated internal phasic DVFs with external phasic and directional DVFs. Principle component analysis is then applied to the composite matrix to extract principal motion characteristics, and generate model parameters to correlate the internal-external motion. The proposed model is evaluated on a 4D NURBS-based cardiac-torso (NCAT) synthetic phantom and 4DCT images from five lung cancer patients. For tumor tracking, the center of mass errors of the tracked tumor are 0.8(±0.5)mm/0.8(±0.4)mm for synthetic data, and 1.3(±1.0)mm/1.2(±1.2)mm for patient data in the intra-fraction/inter-fraction tracking, respectively. For lung tracking, the percent errors of the tracked contours are 0.06(±0.02)/0.07(±0.03) for synthetic data, and 0.06(±0.02)/0.06(±0.02) for patient data in the intra-fraction/inter-fraction tracking, respectively. The extensive validations have demonstrated the effectiveness and reliability of the proposed model in motion tracking for both the tumor and the lung in lung cancer radiotherapy.

## Introduction

Organs and tumors in the thoracic and abdominal region can move and deform significantly due to respiration^1^. The respiration-induced tumor motion can be up to 3 cm in the superior-inferior (SI) direction^2^ and 2–4 mm in the anterior-posterior (AP) direction^3^. Respiration motion-induced translation, rotation, and deformation of the tumor and surrounding organs at risk (OARs) can cause significant geometric and dosimetric errors. Gierga *et al*.^4^ reported that the planned target dose-volume histogram (DVH) was significantly degraded where the received CTV dose was reduced by 2–28% with tumor motion of 7.4 mm and 3.8 mm in the SI and AP directions, respectively. In a 4D Monte Carlo study, 3–5% dose differences (9.3 Gy tumor under-dosage) was observed between a 3DCT plan and a 4DCT plan where respiratory motion was considered^5^. Even with additional planning target volume (PTV) margin compensations for breathing motion, dose deviation of PTV D95 can be up to 26% for fractional dose and 14% for total dose with tumor motion^6^.

Respiration motion, on the other hand, can be utilized as an additional degree of freedom besides conventional 3D spatial domain to achieve 4D optimized treatment plan with greater OAR-sparing while maintaining PTV coverage and delivery efficiency^7^. The primary step to take advantage of respiration motion is to accurately track both the tumor and OARs motion. Many motion tracking strategies have been investigated in the past two decades. Some have been already successfully implemented in clinics^8–12^. Intuitively, directly imaging of tumor and OARs to obtain real-time positions is the most accurate tracking method^13–16^. However, real-time imaging techniques often have their own limitations. For instance, the X-ray fluoroscopy has low image contrast in soft tissue^15,16^. MRI can provide high-quality soft tissue images and is promising for tumor and OAR tracking^13,14^; however, it is expensive and not widely available in clinics yet. While the clinically available MRI guidance radiotherapy units only have 2D planar tracking capability^14,17–19^. Instead of tracking tumor and OAR directly, tracking of the surrogates, e.g., implanted fiducial markers, is a preferable alternative. Studies have shown that fiducial tracking can achieve tumor localization accuracy of up to 0.4 mm^20–22^. However, the popularity of fiducial tracking is impeded by its invasiveness^23^ and possible fiducial migration^24^. Furthermore, fiducial tracking by X-ray fluoroscopy may introduce extra imaging dose^20^.

A more clinical practical tracking approach is achieved by constructing a correlation model to correlate the internal tumor/OARs motion and the external surface surrogate signals^2,25^. The internal-external correlation model, either linear or nonlinear, is usually established before treatment and renewed periodically by acquiring updated external and internal motion information^8–12,26–29^. The Cyberknife Synchrony system^8–11^, the Brainlab ExacTrac system^12,29^ and the Vero system (Brainlab, MHI)^30,31^ are three representative clinical applications. The Cyberknife tracks tumor motion based on a model defined between the external motion and internal markers. This model needs to be updated based on X-ray snapshots frequently^32^. The ExacTrac system can correct patient rigid positioning errors by combining continuous optical infrared tracking signal with X-Ray verification of the internal position. The Vero system achieves dynamic tumor tracking by monitoring the implanted markers with two orthogonal kilovoltage (kV) X-ray images^30^. All the above three systems will introduce extra imaging dose to the patient.

Recent studies have demonstrated the feasibility of correlating the internal motions with external motions detected by respiratory surrogates on the surface^33–37^. More recently, with the development of real-time 3D surface imaging system, researchers have developed more comprehensive internal-external correlation models. For example, McClelland *et al*.^35^ proposed a correlation model for inter-fraction tracking by obtaining internal motion from deformable registration of Cine 4D-CT images and related it to a respiratory surrogate signal derived from the 3D skin surface. The extracted external surrogate signal in their method was one-dimensional which tends to underestimate the complex breathing induced motions. Martin *et al*.^34^ built a surrogate-driven motion model for lung tumor motion tracking from image data acquired by cone-beam CT(CBCT) scan. It was reported that the tracking error was reduced to <2.5 mm in either SI or transverse directions. However, the inherently limited field of view of CBCT scanning protocol only allows for tracking a limited region of interests (ROIs) instead of the entire internal volumes. Fayad *et al*.^37^ developed a patient-specific respiratory motion model using the surface surrogate signal on several ROIs between the xiphoid and umbilicus to track the deformation vector fields(DVFs) of the entire CT volume. Tracking accuracy of <2 mm in thirteen anatomical landmarks was achieved, which is superior over conventional methods using the phasic, amplitude, or phasic & amplitude as external surrogates. Fassi *et al*.^36^ also proposed a similar tracking model by correlating internal motion with three external surface surrogates (i.e., the baseline, amplitude and phase extracted from external surface DVFs). Tracking accuracy of 0.7–2.4 mm was demonstrated on the CBCT projections. Since only limited external surrogates were used, the external motion was likely oversimplified and might not be sufficient for internal-external correlation modeling.

In this study, we proposed an internal motion estimation approach by surface mesh matching and internal-external motion correlation modeling by using estimated internal phasic DVFs and external phasic and directional DVFs. The effectiveness of the proposed model is demonstrated by extensive validations on a 4D NURBS-based cardiac-torso (NCAT) synthetic phantom and five clinical lung cancer cases. A preliminary version of this work has been reported in an abstract^38^.

## Methods and Materials

### Ethics statement

This retrospective patient study was approved by Human Research Protection Program Office (HRPPO)/Institutional Review Board (IRB) of The University of Texas Southwestern Medical Center. All methods in this study were conducted in accordance with the relevant guidelines and regulations. Considering that this is not a therapeutical treatment study, our institutional review board waived the need for obtaining written informed consent from the participants.

### Surface meshing

In this study, external and internal motions were represented by deformation of the external surface and internal organ surface, respectively. The surfaces used for modeling and validation were extracted from the 4DCT, on which the contours were first delineated and then the superior and lateral portion of the body contours, and the internal organ contours were converted to meshes using a particle-based surface meshing approach^39^. Given the contour points of the segmented organ masks (or particles in this algorithm), a high quality isotropic triangular surface meshing can be obtained by solving an inter-particle energy function with the quasi-Newton L-BFGS optimizer. The algorithm implementation details have been described by Zhong *et al*.^39^.

### Motion and deformation tracking model

The internal and external DVFs were denoted as *I*_*j*_ and *S*_*j*_ to characterize the motion of internal organs and external surface on phase *j* of the planning 4DCT images. The *I*_*j*_ and *S*_*j*_ can be represented as Equations (1) and (2).

1 $${I}_{j}=[{I}_{1,x,j},{I}_{1,y,j},{I}_{1,z,j};\ldots ;{I}_{M,x,j},{I}_{M,y,j},{I}_{M,z,j}],$$

2 $${S}_{j}=[{S}_{1,x,j},{S}_{1,y,j},{S}_{1,z,j};\ldots ;{S}_{N,x,j},{S}_{N,y,j},{S}_{N,z,j}].$$

Here, *x*, *y*, *z* represented three cartesian coordinates, *M* was the number of vertices of the internal organ surface, and *N* was the number of small patches on the external surface. Here, *N* = 154 patches (14 × 11, 14 in the SI direction and 11 in the ML direction) were uniformly extracted from the external surface via the strategy detailed in a previous work^33^. Both *I*_*j*_ and *S*_*j*_ were estimated by registering the surface points on phase *j* (*j* ∈ [1,10]) of the 4D planning CT to those on a middle position (MidP) CT, which is a time-averaged CT image representing mean position of patient’s anatomy in the breathing cycle^40^. The DVFs (*S*_*n*,*x*,*j*_, *S*_*n*,*y*,*j*_, *S*_*n*,*z*,*j*_) on patch *n* were calculated by averaging the DVFs on vertices inside the corresponding patch. Surface point registration was accomplished using a recent developed local topology preserved non-rigid point matching algorithm(TOP-DIR)^41^. Unlike internal motion, which was the combination effect of respiration, heart beating and organ deformation, the external surface motion is mainly caused by breathing. However, respiration induced motion may diminish when propagating from internal to external, and is weakly reflected on the external surface. To capture subtle external motion difference between different breathing process (e.g., from inhale to exhale vs. from exhale to inhale), we defined an *A*_*j*_ to describe *respiration-induced directional external motion* between consecutive respiratory phases:

3 $${A}_{j}=[{A}_{1,x,j},{A}_{1,y,j},{A}_{1,z,j};\ldots ;{A}_{N,x,j},{A}_{N,y,j},{A}_{N,z,j}].$$

*A*_*j*_ was calculated by subtracting the DVFs *S*_*j*−1_ from DVFs *S*_*j*_. Vector *d*_*j*_ was constructed by combining *I*_*j*_, *S*_*j*_ and *A*_*j*_ to describe the internal and external motion on phase *j*:

4 $${d}_{j}=[{I}_{1,x,j},{I}_{1,y,j},{I}_{1,z,j},\ldots ,{I}_{M,z,j},\,{S}_{1,x,j},{S}_{1,y,j},{S}_{1,z,j},\,{A}_{1,x,j},{A}_{1,y,j},{A}_{1,z,j},\ldots ,{S}_{N,x,j},\ldots ,{A}_{Nzj}]$$

The internal-external motion pattern for all phases was described by a composite matrix:

5 $$D=[{\tilde{d}}_{1},{\tilde{d}}_{2},\ldots ,{\tilde{d}}_{J}],$$

where ${\tilde{d}}_{j}={d}_{j}-\bar{d},\,\bar{d}=\frac{1}{J}\sum _{j}{d}_{j},\,J=10$. The principle component analysis (PCA) was used to extract motion characteristics from *D*. Instead of calculating the eigenvalues and the corresponding eigenvectors of the covariance matrix *DD*^*T*^ directly with a standard PCA procedure, we adopted the approach of Fayad *et al*.^37^ to reduce computational expense. Let *X*and *λ* be the eigenvectors and eigenvalues of matrix *D*^*T*^*D*,

6 $${D}^{T}DX=\lambda X$$

multiplying *D* on both side of Equation (6) leads to:

7 $$D{D}^{T}(DX)=\lambda DX$$

Equation (7) indicates that *DX* and *λ* are the eigenvectors and corresponding eigenvalues of the covariance matrix *DD*^*T*^. Note that *λ* was the eigenvalues of both *D*^*T*^*D* and *DD*^*T*^, and computation of eigenvalues and corresponding eigenvectors from *D*^*T*^*D* was more efficient (*D*^*T*^*D*was with size *J* × *J*). Finally, the internal-external motion at time *t* can be approximated as a weight sum of the obtained eigenvectors *E* = [*e*_1_, …, *e*_*K*_] of the largest *K*(*K* ≤ *J* − 1)eigenvalues as the Equation (8) below, where *W* was the corresponding weight.

8 $$d(t)\approx \bar{d}+\tilde{d},\tilde{d}=\sum _{k=1}^{K}{w}_{k}(t){e}_{k}=EW$$

Then, $\tilde{d}$ was split into $\tilde{I}\approx {E}_{I}W$, $\tilde{S}\approx {E}_{s}W$, where *E*_*I*_ (size 3*M* × *K*) was constructed from the first 3*M* rows of *E* and *E*_*S*_ (size 6*N* × *K*) was constructed from the rest 6*N* rows of *E*. Essentially, $\tilde{I}$ and $\tilde{S}$ were the internal and external DVFs, and *E*_*I*_ and *E*_*S*_ correspond to the internal and external components of the eigenvectors. By assuming *E*_*S*_ was invertible and eliminating the unknown weight matrix *W*, the internal DVFs $\tilde{I}$ can be predicted using the external DVFs $\tilde{S}$ as:

9 $$\tilde{I}(t)\approx {E}_{I}{E}_{S}^{-1}\tilde{S}(t)=B\tilde{S}(t).$$

where *B* (size 3*M* × 6*N*) was a matrix correlating the internal and external motion.

### Quantification of tracking accuracy

The accuracy of the proposed model was assessed by comparing the tracked internal organ contours with the manually delineated ground truths. Four similarity metrics were used including the center of mass (COM) error, the Dice’s coefficient (DC)^42^, the percent error (PE)^43^ and the Housdourf’s distance (HD)^44^. Given two volumes *A* (manual contoured volume/ground truth) and *B*(predicted volume), and their corresponding boundary points $\vec{A}=\{{a}_{1},\ldots {a}_{p}\}$ and $\vec{B}=\{{b}_{1},\ldots ,{b}_{q}\}$. COM error was defined as the 3D Euclidean distance between the mass center of ground truth volume *A* and that of predicted volume *B*, to measure the tracking accuracy of the motion trajectory. DC, PE and HD were used to measure the agreement between the predicted tumor and organ contours and the manual delineated ground truths. The COM, DC and PE were defined as:

10 $${\rm{COM}}=||c(A)-c(B)||,$$

11 $$DC=2(A\cap B)/(A+B),$$

12 $$PE=(A\cup B-A\cap B)/A.$$

In Equation (10), *c*(*A*) and *c*(*B*) represented the mass center coordinate of volume *A* and *B* and ||·|| was the *L*_2_ norm. DC ranges from 0 to 1, corresponding to the worst and the best agreement, respectively. PE ranges from 0 to infinity, with 0 represents the best agreement. The HD was defined as:

13 $$HD=\,{\rm{\max }}(h(\vec{A},\vec{B}),h(\vec{B},\vec{A})),$$

where $h(\vec{A},\vec{B})={{\rm{\max }}}_{a\in \overrightarrow{A}}{{\rm{\min }}}_{b\in \overrightarrow{B}}\Vert a-b\Vert$. The ground truth organ masks were contoured by an experienced physician. Better tracking results were indicated by lower COM, PE, and HD, or higher DC.

The developed model using the phasic DVFs and directional DVFs on the entire external surface (including all discrete surface patches, termed as SurMod) was compared with 1) using phasic DVFs (without directional DVFs) on partial external surface (10 selected patches, termed as RoiMod)^37^, and 2) using phasic DVFs (without directional DVFs) on the entire external surface (termed as SurphaMod).

Independent samples Kruskal-Wallis (K-W) test was adopted for organ tracking performance comparisons among RoiMod, SurphaMod, and SurMod. Inter-group comparisons were performed with Mann-Whitney tests. K-W tests and one-way analysis of variance (ANOVA) were conducted for non-parametric and parametric data, respectively, to analyze tracking performance difference. All statistical analyses were implemented using SPSS 19.0 software (SPSS Inc., Chicago, IL), and the statistical significance level was set at *p* = 0.05. For multiple comparisons, the *p*-value was adjusted accordingly using the Bonferroni correction method for individual comparison tests in SPSS.

### Synthetic cases

4DCT images with five breathing cycles (Cycles 1–5) simulated by using the 4D NCAT phantom^45^ were employed for evaluation. As detailed in Table 1, the changes of breath period, amplitude and the atrophy of tumor response to the treatment were synthesized in the respiration motion in cycles 1–5. The tumor motions in the ML (Medio-Lateral), AP and SI directions for each respiratory cycle were given by:

14 $$\begin{array}{l}{H}_{ML}=H/10\ast sin(\frac{2\pi }{T}(t-\frac{1}{2}T))\\ {H}_{AP}=H/10\ast (sin(\frac{2\pi }{T}(t-\frac{1}{4}T))+1)\\ {H}_{SI}=H/2\ast (sin(\frac{2\pi }{T}(t-\frac{1}{4}T))+1)\end{array},$$

where *H* was the maximal amplitude of the surface motion in AP direction, *T* was the respiratory period, *H*_*ML*_, *H*_*AP*_ and *H*_*SI*_ were the motion trajectories of the tumor centroids in the ML, AP and SI directions, respectively. The resolution and voxel size of the synthetic cases were 256 × 256 × 120 and 2.0 mm × 2.0 mm × 2.5 mm.

**Table 1:** Simulation parameters of the NCAT phantom.

Cycle #	Period (s)	Maximal Amplitude (mm)	Tumor Diameter (mm)
1	5	12	30
2	4.5	10	30
3	5.5	14	30
4	4	8	20
5	6	16	20

In this NCAT phantom evaluation, Cycle 1 was used for motion modeling. The established correlation model was then applied for motion tracking, where Cycles 2 and 3 were used for intra-fraction validation (with different periods but same tumor diameter), and Cycles 4 and 5 were used for inter-fraction validation (with different periods and different tumor diameter).

### Clinical cases

Clinical 4DCT images from five lung cancer patients (4 males and 1 female, ages range from 53 to 78 with median of 63) were collected for validations (Table 2). All the 4DCT images were acquired on a Brilliance Big Bore-16 (Philips) CT scanner. The 4DCT images were sorted into 10 phases. Patients 1–3 have one set 4DCT image, and patients 4 and 5 have two sets 4DCT images. Both *intra-4DCT* and *inter-4DCT* evaluations were conducted. For intra-4DCT evaluation, the leave-one-out method was used, i.e., nine out of ten phases were used for modeling and the left one phase for motion tracking validation. For inter-4DCT evaluation, 10 phasic 4DCT images from one set were used for modeling while those from the other set for tracking validation.

**Table 2:** Characteristics of the clinical lung cancer cases.

Patient # (4DCT set #)	Resolution	Voxel Size (mm × mm × mm)	Evaluation Case #	
			*Intra-*	*Inter-*
1	512 × 512 × 142	1.0 × 1.0 × 3.0	1	
2	512 × 512 × 149	1.0 × 1.0 × 3.0	2	
3	512 × 512 × 104	1.0 × 1.0 × 2.5	3	
4(1)	512 × 512 × 88	1.4 × 1.4 × 5.0	4	8, 9
4(2)	512 × 512 × 100	1.1 × 1.1 × 5.0	5	8, 9
5(1)	512 × 512 × 93	1.2 × 1.2 × 5.0	6	10, 11
5(2)	512 × 512 × 101	1.2 × 1.2 × 5.0	7	10, 11

### Data availability

The datasets generated during and/or analyzed during the current study are available from the corresponding author on reasonable request.

## Results

### NCAT phantom

**Figure 1:**
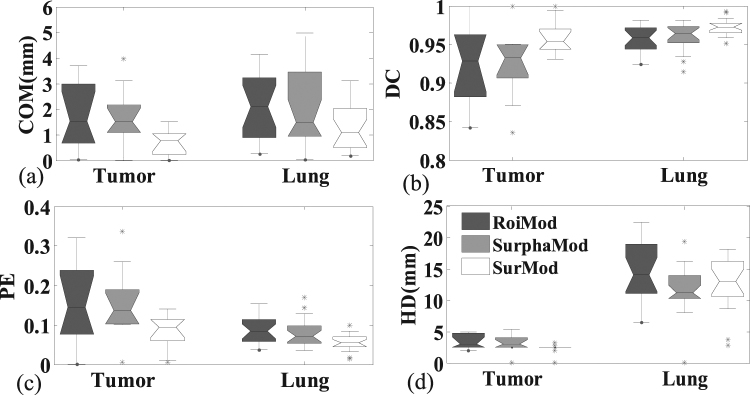
The tracking accuracy comparisons of the RoiMod, SurphaMod, and SurMod using Cycle 2 of the NCAT phantom. The boxes run from the 25th to 75th percentile; the two ends of the whiskers represent the 5% and 95% percentiles of the data, the horizontal line in the box represents the median values, and the stars represent outliers, respectively.

The quantitative comparisons, in terms of COM error, DC, PE and HD, between the RoiMod, SurphaMod, and SurMod are illustrated in Fig. 1. Only slight improvement is observed when comparing the SurphaMod with the RoiMod, while the proposed SurMod achieved the highest tracking accuracy for all the four metrics except for the HD in the lung tracking. The limited improvement of SurphaMod over RoiMod can be explained by the almost consistent surface motion pattern observed in the NCAT phantom, where employing the entire surface does not necessarily provide additional information in motion modeling. While further adding directional DVFs, as demonstrated by the SurMod, can offer more useful motion information in modeling the internal-external correlation.

**Figure 2:**
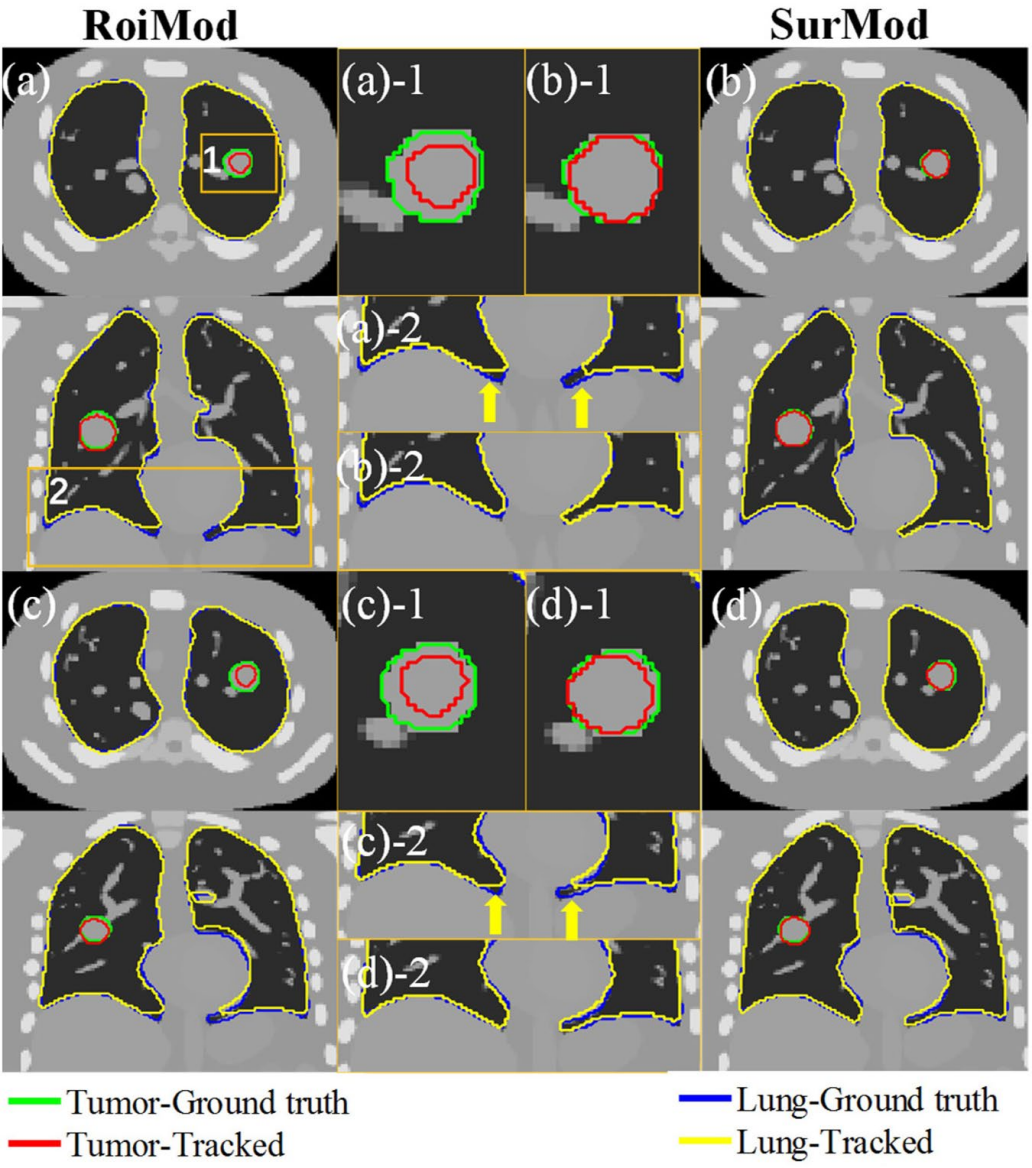
Tumor and lung tracking at the end of inspiration (**a**) and (**b**) and at the end of expiration (**c**) and (**d**) in one breathing cycle (Cycle 2) on the NCAT phantom. The zoom-in views (middle column) are extracted from the ROIs as labeled in (**a**).

**Figure 3:**
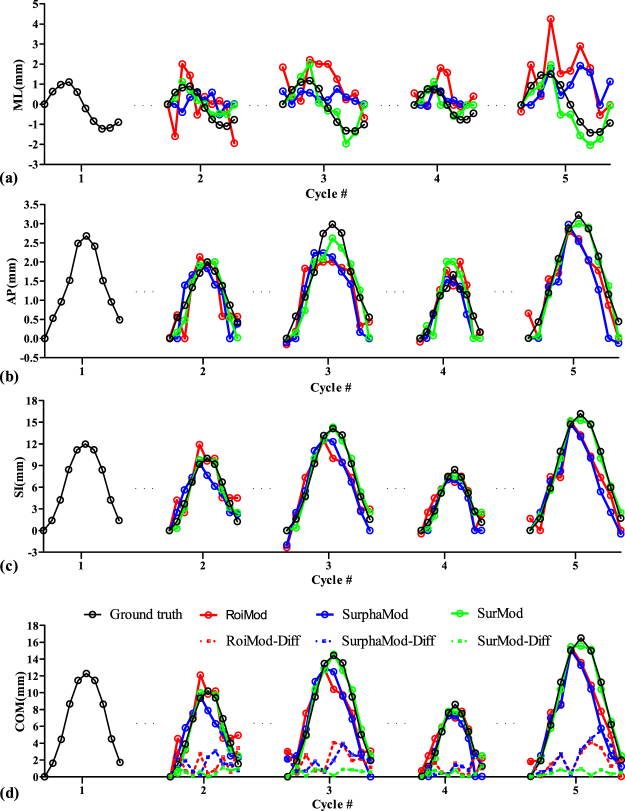
Tumor trajectories in the ML (**a**), AP (**b**), SI (**c**) directions, as well as the COM trajectories and COM differences (**d**) of the NCAT phantom.

The advantage of utilizing complete surface and directional DVFs in motion modeling is visualized as an example case shown in Fig. 2, where the SurMod and the RoiMod are compared at the end of inspiration (Fig. 2(a), (b)) and at the end of expiration (Fig. 2(c), (d)). It is observed that the SurMod can produce more accurate The RoiMod and SurphaMod both failed in tracking the tumor motion in the ML direction (Fig. 3(a)), even though the simulated motion is relatively small (~3 mm in the ML direction). In contrast, the proposed SurMod is able to capture those subtle changes in the ML direction tracking than the RoiMod for both the tumor and the lung, especially in the apex of the lung (Fig. 2(a–d)-2).

Figure 3 shows the intra-fraction and inter-fraction tracking comparisons in the ML, AP and SI directions as well as the COM trajectories and COM differences. The RoiMod and SurphaMod both fail in tracking the tumor motion in the ML direction (Fig. 3(a)), even though the simulated motion is relatively small (~3 mm in the ML direction). In contrast, the proposed SurMod is able to capture those subtle changes in the ML direction, and yields superior tracking accuracy in all directions (Fig. 3(a), (b), (c)). The largest COM errors are 4.1 mm, 5.3 mm and 1.3 mm for the RoiMod, SurphaMod, and the SurMod, respectively. The mean errors in the ML, AP, SI directions of the RoiMod are 1.2(±0.8) mm, 0.4(±0.3) mm, 1.7(±1.2) mm, respectively. The SurphaMod yields similar results with a mean error of 1.1(±0.8) mm, 0.4(±0.3) mm and 1.6(±1.4) mm. In comparison, the proposed SurMod achieves best tracking accuracy with a mean error of 0.4(±0.3) mm, 0.3(±0.2) mm and 0.5(±0.4) mm in the above three directions. In general, the SurMod decreases the mean COM error from 1.6(±1.2) mm and 1.7(±1.4) mm to 0.8(±0.4) mm in tumor tracking when compared with the RoiMod and SurphaMod.

**Figure 4:**
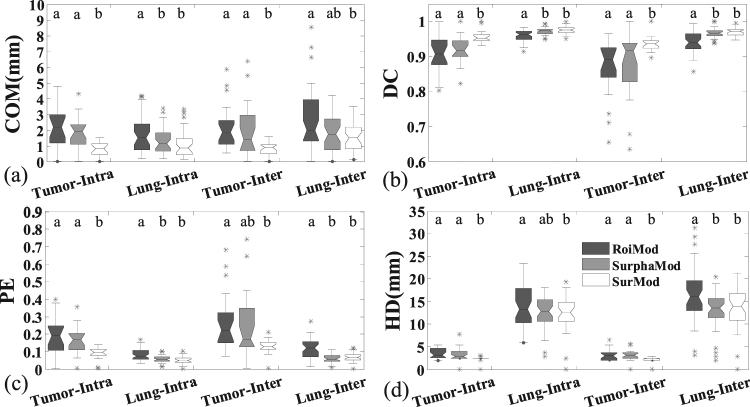
The quantitative comparisons between the RoiMod, SurphaMod, and SurMod in intra-fraction tracking (Cycles 2, 3) and inter-fraction tracking (Cycles 4, 5) on the NCAT phantom. The meanings of the symbols in this figure are the same as in Fig. 2. The letters above each box indicate whether a statistically significant difference exists between any two tracking models. With different letters indicates statistical significance while the same letters for statistical insignificance.

The quantitative evaluations on the synthetic phantom are illustrated in Table 3 and Fig. 4. For both intra-/inter-fraction tracking, the SurphaMod achieves significant improvements over RoiMod in lung tracking accuracy, except for the HD of the intra-fraction tracking and the COM of the inter-fraction tracking. However, no significant improvement is observed in tumor tracking from RoiMod to SurphaMod. In contrast, the proposed SurMod achieves significant improvements over RoiMod in all metrics for both intra-/inter-fraction tumor and lung tracking. Furthermore, the SurMod also achieves significant improvements over the SurphaMod in tumor tracking except for the PE of inter-fraction tracking. After all, both the SurphaMod and SurMod achieve improvements over the RoiMod, and the SurMod performs the best. Quantitatively, the tracking accuracies achieved by the proposed SurMod in terms of COM, DC, PE and HD are 0.8(±0.5)mm/0.8(±0.4)mm, 0.96(±0.02)/0.94(±0.03), 0.09(±0.04)/0.12(±0.05), 2.3(±0.8)mm/2.2(±0.8)mm for tumor, and 1.3(±0.8)mm/1.8(±1.1)mm, 0.97(±0.01)/0.97(±0.01), 0.06(±0.02)/0.07(±0.03), 12.6(±3.5)mm/13.0(±4.3)mm for lung of intra-fraction/inter-fraction tracking on the NCAT phantom, respectively.

**Table 3:** Quantitative comparisons (Mean ± STD(*p*-value)) between the RoiMod, SurphaMod and the SurMod in intra-fraction tracking and inter-fraction tracking for the NCAT phantom.

Intra-fraction tracking (Cycles 2, 3)				
Structures	Quantitative Metrics	RoiMod	SurphaMod (*vs* RoiMod)	SurMod (*vs* RoiMod/SurphaMod)
Tumor	COM(mm)	2.2 ± 1.3	1.8 ± 1.1(>0.05)	0.8 ± 0.5(<**0.001**/**0.003**)
	DC	0.91 ± 0.05	0.92 ± 0.04(>0.05)	0.96 ± 0.02(**0.001**/**0.005**)
	PE	0.19 ± 0.11	0.16 ± 0.08(>0.05)	0.09 ± 0.04(**0.001**/**0.005**)
	HD(mm)	3.5 ± 1.1	3.2 ± 1.8(>0.05)	2.3 ± 0.8(**0.003**/**0.039**)
Lung	COM(mm)	1.7 ± 1.2	1.3 ± 0.8(**0.045**)	1.2 ± 0.9(**0.010**/>0.05)
	DC	0.96 ± 0.02	0.97 ± 0.01(**0.006**)	0.97 ± 0.01(<**0.001**/>0.05)
	PE	0.08 ± 0.04	0.06 ± 0.02(**0.006**)	0.05 ± 0.02(<**0.001**/>0.05)
	HD(mm)	14.1 ± 4.6	12.6 ± 3.5(0.120)	11.8 ± 4.6(**0.018**/>0.05)
**Inter-fraction tracking (Cycles 4, 5)**				
**Structures**	**Quantitative Metrics**	**RoiMod**	**SurphaMod (** ***vs*** **RoiMod)**	**SurMod (** ***vs*** **RoiMod/SurphaMod)**
Tumor	COM(mm)	2.1 ± 1.5	2.0 ± 1.8(>0.05)	0.8 ± 0.4(**0.001**/**0.035**)
	DC	0.87 ± 0.08	0.88 ± 0.10(>0.05)	0.94 ± 0.03(**0.005**/**0.050**)
	PE	0.27 ± 0.17	0.25 ± 0.20(>0.05)	0.12 ± 0.05(**0.005**/0.052)
	HD(mm)	3.3 ± 1.3	3.1 ± 1.5(>0.05)	2.2 ± 0.8(**0.046**/**0.018**)
Lung	COM(mm)	2.7 ± 1.9	1.8 ± 1.2(0.131)	1.5 ± 0.9(**0.014**/>0.05)
	DC	0.94 ± 0.03	0.97 ± 0.02(<**0.001**)	0.97 ± 0.01(<**0.001**/>0.05)
	PE	0.12 ± 0.06	0.06 ± 0.03(<**0.001**)	0.07 ± 0.03(<**0.001**/>0.05)
	HD(mm)	16.5 ± 5.8	13.0 ± 4.3(**0.003**)	13.0 ± 5.3(**0.003**/>0.05)

### Clinical cases

**Figure 5:**
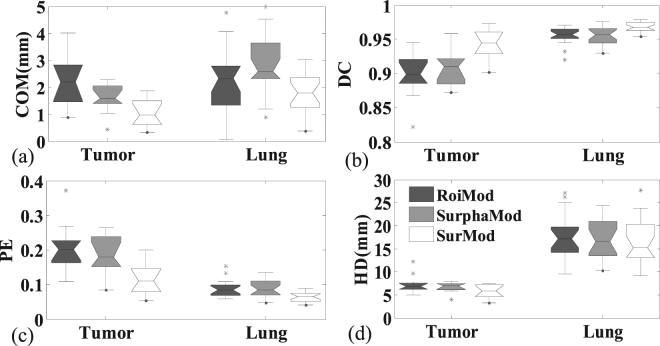
The tracking accuracy comparisons between the RoiMod, SurphaMod and SurMod on the clinical Case 1.

The comparisons of the intra-4DCT tracking for a clinical case (Case 1) are illustrated in Fig. 5. We can see that the SurphaMod is able to produce more accurate tracking results than the RoiMod, though slight inferior COM errors are observed in the lung. Among the three models, the proposed SurMod yields the best results.

**Figure 6:**
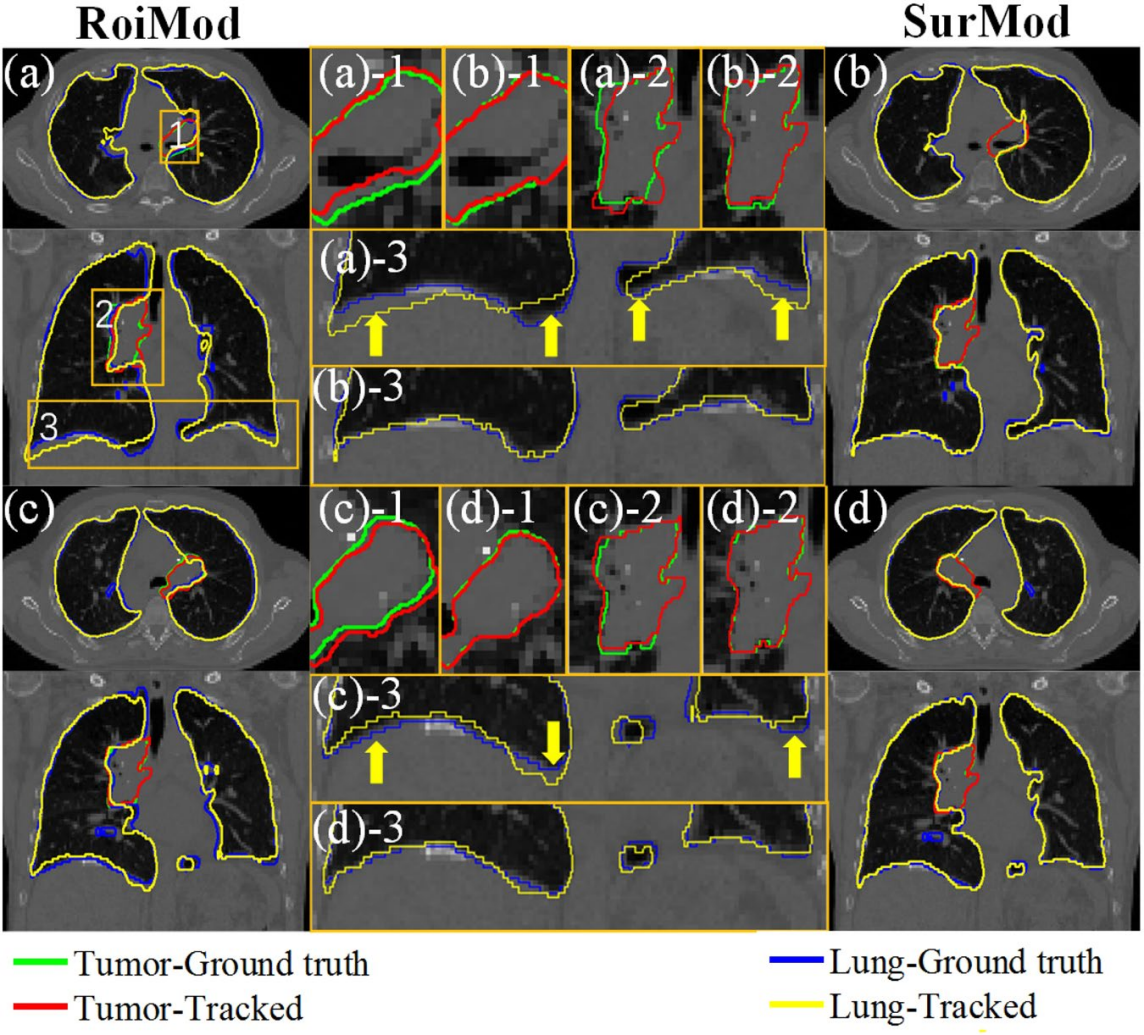
Intra-4DCT tracking of the tumor and lung at the end of expiration (**a**) and (**b**) and at the end of inspiration (**c**) and (**d**) of a clinical patient case (Case 1). The yellow zoom-in views (middle column) are extracted from the ROIs as labeled in (**a**).

Visual comparisons of the intra-4DCT tracking of the clinical case 1 are shown in Fig. 6. More accurate tumor and lung contours are predicted by the SurMod compared with the RoiMod, indicates by better agreements between the tracked contours with the physician delineated ground truths.

**Figure 7:**
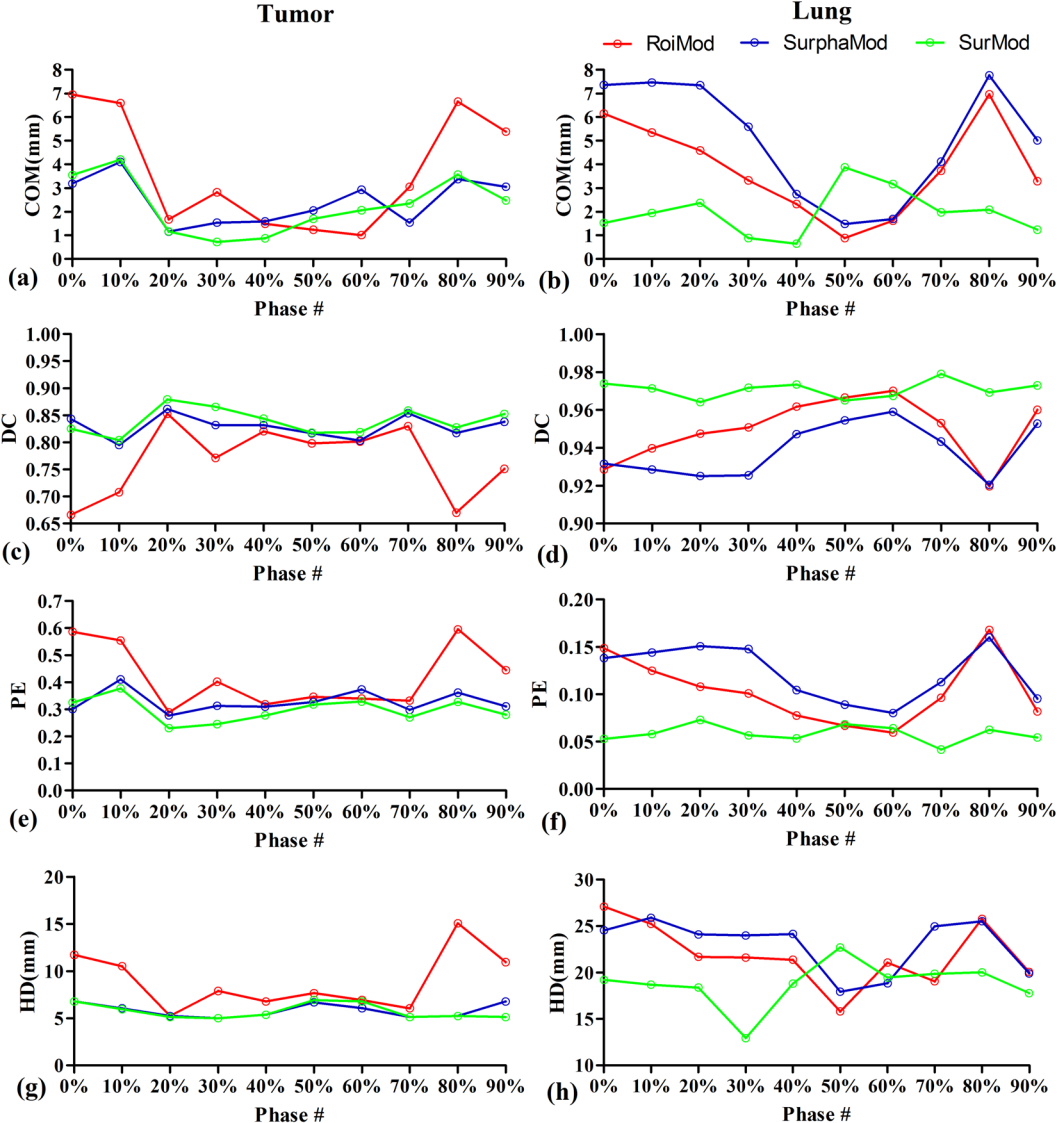
Inter-4DCT tracking accuracy in terms of COM error (**a**,**b**), DC (**c**,**d**), PE (**e,f**), as well as HD (**g**,**h**) for the tumor and lung in a clinical patient case (Case 9). Phase 0% and 50% represent the end of inhale and the end of exhale, respectively.

Figure 7 shows an inter-4DCT tracking example comparison. For the tumor, the RoiMod yields the worst tracking accuracy of COM 3.7(±2.4) mm, DC 0.77(±0.07), PE 0.42(±0.12) and HD 8.9(±3.1), followed by the SurphaMod with COM 2.6(±1.0) mm, DC 0.83(±0.02), PE 0.33(±0.04) and HD 5.8(±0.7) mm. The SurMod achieves best tracking accuracy of COM 2.3(±1.2) mm, DC 0.84(±0.02), PE 0.30(±0.04) and HD 5.7(±0.8) mm. For the lung, similar results are achieved by RoiMod (COM 3.8(±2.0) mm, DC 0.95(±0.02), PE 0.10(±0.04), HD 21.9(±3.4) mm) and SurphaMod (COM 5.1(±2.5) mm, DC 0.94(±0.01), PE 0.12(±0.03), HD 23.0(±2.9) mm), and the proposed SurMod achieves improved accuracies of COM 2.0(±1.0) mm, DC 0.97(±0.01), PE 0.06(±0.01), HD 18.8(±2.4) mm. However, the COM error of SurMod is larger than RoiMod and SurphMod in phase 50% and 60%. The reason for this is that the COM error is a biased metric when the target involves large deformation in motion, especially in scenarios(such as the lung at the end of exhale). The four metrics combined results show the three model behave similarly in phase 50% and 60%.

**Figure 8:**
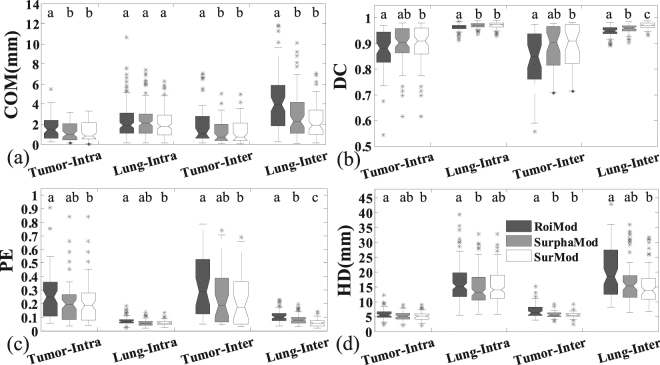
The quantitative comparisons between the RoiMod, SurphaMod, and SurMod in intra-4DCT tracking (Cases 1–7) and inter-4DCT tracking (Cases 8–11) for all the clinical cases. The meanings of the symbols and letters above each box in this figure are the same as in Fig. 4.

The tracking accuracy comparisons for all clinical cases (Cases 1 to 11) are shown in Table 4 and Fig. 8. In the intra-/inter-4DCT tracking, significant improvements are observed from SurphaMod over RoiMod in COM error of intra-/inter-4DCT tracking and HD of the inter-4DCT tracking for tumor, and in all metrics of intra-/inter-4DCT lung tracking except for the COM error of intra-4DCT tracking and the HD of inter-4DCT tracking. The SurMod also achieves significantly better tracking accuracies over RoiMod except for the COM error and HD of intra-4DCT lung tracking. Furthermore, significant improvements also are observed from SurMod over SurphaMod in DC and PE of the inter-4DCT lung tracking. In general, both the SurphaMod and SurMod achieve improvements over RoiMod, but the SurMod yields the best results. Quantitatively, the proposed SurMod improves the tracking accuracy in terms of COM, DC, PE and HD as 1.3(±1.0) mm/1.2(±1.2) mm, 0.90(±0.07)/0.89(±0.08), 0.20(±0.15)/0.23(±0.19), 5.2(±1.5) mm/5.6(±1.1) mm for tumor and 2.1(±1.4) mm/2.3(±1.7) mm, 0.97(±0.01)/0.97(±0.01), 0.06(±0.02)/0.06(±0.02), 15.2(±5.4) mm/15.5(±5.9) mm for lung in the intra-/inter-4DCT tracking on the clinical patient data, respectively.

**Table 4:** Quantitative comparisons (Mean ± STD(*p*-value)) between the RoiMod, SurphaMod and the SurMod in intra-fraction tracking and inter-fraction tracking for all the clinical cases.

Intra-fraction tracking (Cases 1–7)				
Structures	Quantitative Metrics	RoiMod	SurphaMod (*vs* RoiMod)	SurMod (*vs* RoiMod/SurphaMod)
Tumor	COM(mm)	1.6 ± 1.2	1.3 ± 1.0(**0.041**)	1.3 ± 1.0(**0.043**/> 0.05)
	DC	0.87 ± 0.08	0.90 ± 0.07(0.064)	0.90 ± 0.07(**0.038**/> 0.05)
	PE	0.26 ± 0.17	0.21 ± 0.15(0.060)	0.20 ± 0.15(**0.038**/> 0.05)
	HD(mm)	5.7 ± 1.7	5.3 ± 1.6(0.082)	5.2 ± 1.5(**0.049**/> 0.05)
Lung	COM(mm)	2.3 ± 1.8	2.3 ± 1.6(>0.05)	2.1 ± 1.4(>0.05/> 0.05)
	DC	0.96 ± 0.02	0.97 ± 0.01 (<**0.001**)	0.97 ± 0.01 (<**0.001**/> 0.05)
	PE	0.07 ± 0.03	0.06 ± 0.03 (<**0.001**)	0.06 ± 0.02 (<**0.001**/> 0.05)
	HD(mm)	16.1 ± 6.2	14.7 ± 5.7(**0.026**)	15.2 ± 5.4(0.163/> 0.05)
**Inter-fraction tracking (Cases 8–11)**				
**Structures**	**Quantitative Metrics**	**RoiMod**	**SurphaMod (** ***vs*** **RoiMod)**	**SurMod (*vs*** **RoiMod**/**SurphaMod)**
Tumor	COM(mm)	2.1 ± 2.2	1.3 ± 1.3(**0.028**)	1.2 ± 1.2(**0.020**/> 0.05)
	DC	0.84 ± 0.12	0.88 ± 0.09(>0.05)	0.89 ± 0.08(**0.015**/> 0.05)
	PE	0.37 ± 0.33	0.25 ± 0.21(>0.05)	0.23 ± 0.19(**0.032**/> 0.05)
	HD(mm)	7.2 ± 2.6	5.7 ± 1.2(**0.026**)	5.6 ± 1.1(**0.013**/> 0.05)
Lung	COM(mm)	4.3 ± 3.0	3.0 ± 2.3(**0.016**)	2.3 ± 1.7 (<**0.001**/> 0.05)
	DC	0.95 ± 0.03	±0.96 ± 0.02(**0.008**)	0.97 ± 0.01 (<**0.001**/<**0.001**)
	PE	0.11 ± 0.05	0.08 ± 0.04(**0.004**)	0.06 ± 0.02 (<**0.001**/<**0.001**)
	HD(mm)	20.0 ± 8.3	16.7 ± 6.9(0.069)	15.5 ± 5.9 (<**0.001**/> 0.05)

### Efficiency

All experiments in this study were conducted on a CPU platform equipped with 8 GB memory, and the proposed model was coded and implemented on the software platform of MatlabR2011a. The average computational time for motion modeling and prediction were ~9 minutes and ~13 seconds. The computational time was closely related to the number of the generated mesh vertices of the internal organs and external surface. Intuitively, more vertices can depict more anatomical details on organ surface, however, it does not necessarily imply higher registration accuracy^41^. In this study, therefore, the quantity of mesh vertices was empirically set as 1500 to balance the accuracy and efficiency, which was proved to be adequate to yield satisfactory registration result.

## Discussion

As the evaluation results on synthetic NCAT phantom data and clinical data illustrated, the proposed SurMod achieved superior performance over the RoiMod and SurphaMod in the internal organs tracking for lung cancer radiotherapy. It also can be observed that, compared with the performance in clinical cases, the proposed model achieved better in the NCAT phantom. The reasons are two folds: firstly, the breathing pattern is constant in the NCAT phantom but might be irregular in real patient data. The proposed model is established using one set of the 4DCT image with the assumption that change in breathing pattern is small; however, this is an ideal hypothesis which might not happen in more complicated clinical cases. Secondly, since the target shape may vary considerably between different treatment fractions, thus, delineation consistency of the target will introduce another source of error for tracking accuracy evaluation. Compared with the inter-fraction tracking, higher accuracy was seen in intra-fraction tracking. This may attribute to the large anatomical changes between different treatment fractions, even though the model had been adapted by registering the patient anatomy between fractions, the anatomical differences were unlikely to eliminate. Furthermore, the respiration pattern tends to be stable in intra-fraction treatment. However, it may vary considerably between treatment fractions. With effective model update strategies, this tracking accuracy discrepancy between intra-fraction and inter-fraction should be reduced.

The effectiveness of the proposed SurMod attributes to the utilization of motion information from the entire surface, as well as directional respiration-induced DVFs *A*_*j*_. The partial ROIs based model was proved to be superior to the conventional approaches using phasic, amplitude, and phasic & amplitude as external surrogates^37^. However, the location of the ten proposed ROIs were identified from ten patients, which might not be able to generalize to a broader patient cohort because of inter-patient respiration variations. The generally superior performance of the SurphaMod over the RoiMod implied the robustness and effectiveness of using entire surface information. Moreover, benefited from the utilization of directional DVFs *A*_*j*_, the proposed SurMod outperformed SurphaMod in scenarios when large inspiration-expiration differences occurred (e.g., synthetic cases, case 1 and case 9, as results shown in Figs 1, 5 and 7, respectively). In general, compared with the RoiMod^37^, the proposed SurMod improved the mean DC from 0.84/0.95 to 0.89/0.97 for inter-fraction tumor/lung tracking on clinical data (0.015/<0.001) significantly. The mean COM error of 1.2 mm achieved by SurMod is also lower than 2.4 mm reported in a conventional approach using the phases & baseline & amplitude surrogates^36^ in inter-fraction tumor tracking.

For the proposed SurMod, the modeling procedure costs <10 mins in a CPU platform and the motion prediction costs <15 s, where most of the computation time is spent on surface meshes registration. Though 8~10 frames per second (fps) tracking speed^46^ is usually expected in a clinical context for real-time tracking, 10 × or more acceleration, as reported by previous studies^47–50^, is possible and not effort-demanding. This can be achieved by simply coding the current model in parallel in a GPU environment equipped with high-end computational platform.

Though promising results were achieved on synthetic phantom data and clinical patient data, there are many challenges needed to be addressed before applying the proposed model in a clinical setting. Firstly, the proposed model is based on the stable respiration assumption, which may not apply to all patients, especially for those late stage lung cancer patients with poor lung function. Model updating is necessary to adapt the model to a new on-treatment scenario^51,52^, especially for inter-fraction tracking. Therefore, model validation during treatment and a regular model update scheme will be essential for renewing the model established on the pre-treatment 4DCT. The model update can be achieved by registering the patient anatomy on the treatment day with that from the planning 4DCT, which is also used to accommodate the inter-fractional baseline drift in this study. The efficiency of the model update is closely related to the registration algorithm used. The registration process is expected to be done within half a minute with the aid of GPU acceleration^53^. With current technologies, the kV X-ray verification is the most practical tool for the tumor tracking model validation, which is also easy for model update to accommodate the baseline shift estimation. Since the purpose of the proposed model was to track both tumor location and tumor shape, projection images from multiple directions might be required to provide benchmark comparisons of tumor shape in different projection angles.

Furthermore, more complicated circumstances (e.g. phase shifts, baseline drifts and hysteresis, etc.) need to be evaluated for the proposed model. In this study, to evaluate the model’s responses to general respiration motion, primary parameters changes (period, amplitude and tumor shape) were simulated in the synthesized motion in the NCAT phantom study. We did not synthesize more complicated motion, where the reasons are two folds: (1) there are too many possibilities of respiration pattern to be evaluated if more parameters are added in motion simulation. Even if all these possibilities were enumerated, it is still difficult, if not impossible, to realistically synthesize respiration since breathing pattern is far more complicated and diversified in patients; (2) the tracking accuracies responding to above practical issues are closely related to the corresponding solution scheme. For example, aligning the patient anatomy on the treatment day with that defined on modeling is an alternative solution to address inter-fractional baseline drift. The influence in tracking accuracy from the baseline drift is directly related to the registration uncertainties of the adopted registration algorithm, which is beyond the scope of the proposed mathematical model. However, more comprehensive model evaluation and adaption need to be addressed in future studies before successful clinical applications.

Finally, in clinical practice, the external surface is obtained by optical surface imaging devices (e.g. ToF camera and AlighRT etc.), which has certain practical issues such as, image acquisition latency. Latency is a common issue in many commercial surface monitoring systems, and model forward prediction might be a practical way to accommodate this issue. For the proposed model, which was built on external DVFs estimated from DIR of external surface mesh, a forward predicted surface mesh might be needed. As an alternative, the external DVFs can be extrapolated using known phasic external DVFs, or breathing velocity/accelerated velocity calculated from directional external DVFs.

## Conclusion

In this work, we proposed and validated a novel motion-tracking model in lung cancer radiotherapy, which is constructed based on the correlation between the phasic DVFs on the internal organs’ surface and phasic and inter-phasic DVFs on the patient’s external surface. Experimental evaluations were conducted on a synthetic 4D NCAT phantom and 4DCT images from five lung cancer patients. The evaluation results have demonstrated the capability of the proposed model in internal organs tracking with the variance of breath frequency, amplitude, inter-fraction anatomical baseline shift and deformation. The good performances on the evaluation data demonstrated the effectiveness, reliability and accuracy of the proposed model. However, more comprehensive model evaluation and model adaption are needed before adopting the proposed model in a clinical setting.
